# Pre-clinical Evaluation of an Adult Extracoproreal Carbon Dioxide Removal System for Pediatric Application

**DOI:** 10.5195/cajgh.2014.167

**Published:** 2014-12-12

**Authors:** Yerbol Mussin, Richard Jeffries, Denis Bulanin, Zhaksybay Zhumadilov, Farkhad Olzhayev, William Federspiel

**Affiliations:** 1 Center for Life Sciences, Nazarbayev University, Astana, Kazakhstan; 2 McGowan Insitute for Regenerative Medicine, Pittsburgh, USA

**Keywords:** Hemolung, extracorporeal carbon dioxide removal, pediatric ECMO, respiratory support

## Abstract

**Introduction:**

Adult extracorporeal carbon dioxide removal (ECCO2R) systems and pediatric ECMO share the common objectives of having a low blood flow rate and low priming volume while safely maintaining sufficient respiratory support. The Hemolung is a highly simplified adult ECCO2R system intended for partial respiratory support in adult patients with acute hypercapnic respiratory failure. The objective of this work was to conduct pre-clinical feasibility studies to determine if a highly efficient, active-mixing, adult ECCO2R system can safely be translated to the pediatric population.

**Methods:**

14 healthy nonsedated juvenile sheep were used for acute (2 animals) and 7-day chronic (12 animals) in-vivo studies to evaluate treatment safety independently of respiratory related injuries. In all evaluations, we hypothesized that gas exchange capabilities of the Hemolung RAS in this model would be equivalent to the adult configuration performance at similar blood flows - minimum CO2 removal of 50 mL/min at a venous partial pressure of CO2 equal to 45 mmHg. Target blood flow rates were set to a minimum of 280 mL/min. Swan Ganz catheters were used under general anesthesia in the two acute subjects to evaluate blood gas status in the pulmonary artery.

**Results:**

The Hemolung RAS was found to have adequate gas exchange and pumping capabilities for full respiratory support for subjects weighing 3 – 25 kg. The Hemolung device was estimated to provide a partial respiratory support for subjects weighing 27 – 34 kg. The seven-day studies in juvenile sheep demonstrated that veno-venous extracorporeal support could be provided safely at low flows with no significant adverse reactions related to device operation.

**Conclusion:**

The study outcomes suggest the potential use of the Hemolung RAS in a veno-venous pediatric configuration to safely provide respiratory support utilizing a significantly less complex system than traditional pediatric ECMO.

